# A Staged Surgical Treatment Outcome of Type 3 Open Tibial Fractures

**DOI:** 10.1155/2014/721041

**Published:** 2014-04-15

**Authors:** Ahmet Aslan, Emin Uysal, Ahmet Özmeriç

**Affiliations:** ^1^Departments of Orthopedics and Traumatology, Afyonkarahisar State Hospital, Orhangazi Mah. Nedim Helvacıoglu Caddesi No. 73, 03100 Afyonkarahisar, Turkey; ^2^Department of Emergency Medicine, Bağcılar Education and Research Hospital, Istanbul, Turkey; ^3^Departments of Orthopedics and Traumatology, Ankara Education and Research Hospital, Ankara, Turkey

## Abstract

*Aim*. In these case series which are about type 3 open tibial fractures formed with three different high energy trauma etiologies in different parts of tibia. We aimed to assess our three-stage treatment approach and discuss final results of our elective surgery management with three different fixation methods. *Patients and Methods*. We assessed 19 patients with type 3 open tibial fractures between 2009 and 2012. Our treatment protocol consisted of three stages. Early intervention in operating room, which including vascular repairs or soft tissue closure, was done if necessary. Definitive surgery was performed using internal or external fixation in the first 15 days. Patients were followed up for at least one year. Last conditions of all our cases were evaluated according to modified Johner and Wruhs criteria. *Results*. Nine cases were type 3A, seven cases were type 3B, and three cases were type 3C in terms of fracture typing. All patients were followed up for at least one year and mean follow up time was 15 months. In terms of functional and clinical outcome, six cases were evaluated as excellent, eight cases as good, two cases as fair, and three cases as poor. *Discussion*. Staged treatment option in type 3 open tibial fractures seems to be a good method in reducing complication and achieving the best result. We think that definitive staged treatment protocol including internal fixation with plating or intramedullary nailing (IMN) of the fractures is a reliable method, especially to avoid complications as a result of external fixator and to provide patient rapport.

## 1. Background

Tibial fractures, in which approximately 15% of all adult fractures are seen, frequently caused by direct or indirect traumas due to slimness of cutaneous and subcutaneous tissues which are anterior of tibial shaft. Epidemiologic studies have shown that compound fractures are 23.5% of all tibial shaft fractures. In treatment of compound tibial fractures, rate of experiencing complications like infection, delayed union, or nonunion is high because of weak perfusion and high density of cortical bone material [1–4]. Especially Gustillo-Anderson type 3 open tibial fractures are caused by high energy traumas and are frequently accompanied by serious complications like amputation, infection, nonunion, malunion, and soft tissue losses. Use of new generation antibiotics, adequate irrigation and debridement, and new methods on fixation techniques has reduced these complications. But, because most of them are formed as a result of high energy traumas and may contain vascular-neural lesions or vast soft tissue damages, management of type 3 open fractures has still been challenging for orthopedic surgeons and prognosis can be affected by complications like compartment syndrome, osteomyelitis, nonunion, or even amputation [5–7].

Treatment methods on open tibial fractures depend on characteristics of fracture, age, general condition of patient, situation of surrounding soft tissue, and circulatory properties. The method which will be preferred has to allow maximum functional restoration for extremities and has to enable optimal bone alignment and length. Most important elements affecting prognosis positively in compound tibial fractures are early treatment, providing enclosure on circulation and soft tissue, infection prophylaxis, fixation of the fracture with an optimal surgical technique, and effective rehabilitation program. In treatment of compound tibial fractures which are classified according to current soft tissue damage, maintaining reliable fixation of fracture with minimal soft tissue damage, after providing integrity of damaged soft tissue, will affect outcome positively [8–12].

In light of various scoring systems, along with extremity saving interventions, primary amputation is an option in especially types 3B and 3C fractures; however, it attracts attention to that, nowadays, most of discussions focus on type of fixation material. Many authors have applied external or internal fixation methods in type 3 compound tibial fractures. While treatment with external fixation in type 3 compound tibia fractures is generally a recognised technique, nowadays, there are reports which suggests internal fixation in these types of fractures [13–17].

In these case series which are about type 3 open tibial fractures formed with three different high energy trauma etiologies in different parts of tibia (proximal, medial, and distal), we aimed to assess our three-stage treatment approach and discuss final results of our elective surgery management with three different fixation methods.

## 2. Patients and Methods

We assessed 19 patients with type 3 open tibial fractures between 2009 and 2012. Fractures are classified according to AO/OTA and open fractures are typed according to Gustilo-Anderson classification.

First interventions to patients were debridement, irrigation, and splint casting in emergency conditions. This first stage was performed immediately in the emergency room. All patients were given tetanus prophylaxis and infection prophylaxis with antibiotics for type 3 compound fractures. Cefazolin 1 g three times a day and gentamycine 80 mg two times a day were applied for antibiotic prophylaxis during 72 hours. After that in early period, radical debridement-irrigation with plenty of SF and temporary external fixation with external fixator were achieved in operating room conditions. The second-stage procedure was performed during the initiation after 48 hours. If vascular repairs or closure of soft tissue was necessary, it would be performed. In the third stage, during the first 15 days (3–15 days, mean 9 days), patients would undergo surgery with internal fixation (IMN/plate-screw) or external fixation (Ilizarov) unless a sign of infection was seen clinically or with laboratory results or discharging in the bottom of schanz nails. Patients were allowed to walk with 2 crutches without enforce heavy on lower extremities in early periods. Patients were followed up for at least a year. Last conditions of all our cases were evaluated according to modified Johner and Wruhs' [18] criteria.

## 3. Results

The mean age was 35.3 years. Thirteen patients were male and six were female, and fourteen of fractures were in right and five of them were in left tibia. Cases according to fracture classification (AO/OTA) are shown in Table 1. Nine cases was type 3A, seven was type 3B, and three cases were type 3C in terms of fracture types. Results according to the type of fracture were shown in Table 2. All patients were followed up for at least a year and mean followup time was fifteen months. In terms of functionality and clinical outcome, six cases were evaluated as excellent, eight cases as good, two cases as fair, and three cases as poor (Table 1).

**Figure 1:**
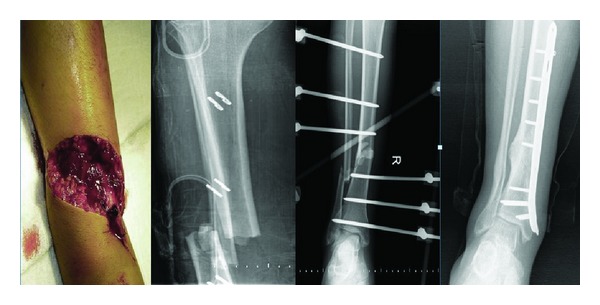
Case 13: AO/OTA: 42B2, GA: 3A, 28-year-old male, traffic accident, elective surgery (third stage): plate-screw, 14-month followup, X-ray: full union.

**Figure 2:**
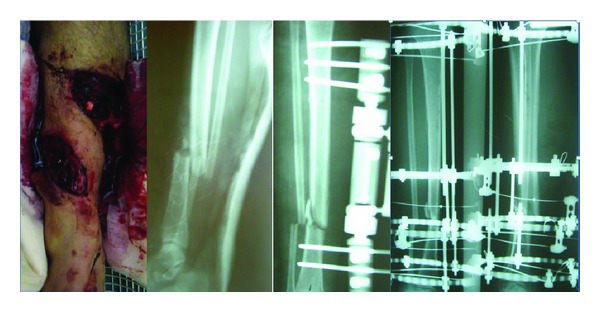
Case 7: AO/OTA: 42B2, GA: 3B, 48-year-old male, traffic accident, elective surgery (third stage): Ilizarov, 10-month followup, X-ray: there is no adequate callus.

**Figure 3:**
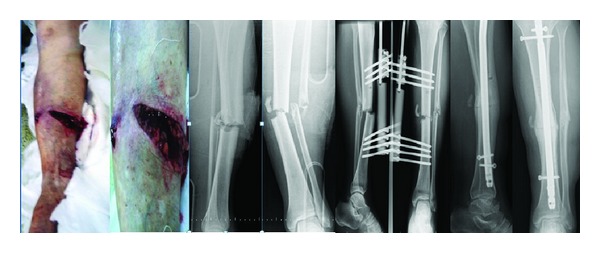
Case 11: AO/OTA: 42B2, GA: 3C, 43-year-old female, traffic accident, second stage: vessel repair, third stage: IMN, 15-month followup, X-ray: full union.

One of two cases with type 3B open fracture needed loose flap and the other case needed soft tissue closure. Popliteal artery was repaired in one case with type 3C open fracture, posterior tibial artery was repaired in another case and saphenous vein repair were made in the other case with type 3C fractures; each vein repair was made in a different case. In definitive surgery stage, radial fracture accompanied one case with type 3A open fracture and a calcaneal fracture accompanied a different case with type 3A open fracture were treated. In third stage of treatment; eight cases undergone intramedullary nailing surgery, six cases were fixated with external fixator, and five cases with plate-screw. Applied in the third stage of definitive treatment results were shown in Table 2. Examples from our cases are shown in Figures 1, 2, and 3.

## 4. Discussion

open fractures are an important reason for mortality and morbidity among all musculoskeletal injuries and a socioeconomical problem for society. Behrens and Searls [19] emphasized that every year two cases of 1000 injuries were compound tibial fractures and this rate was greater than 0.2% in developing countries. Although treatment approaches for compound fractures have shown progression in recent years, especially Gustillo-Anderson, type 3 open fractures remain to be one of the important problems in orthopedic surgery because of high energy trauma, vasculoneural lesions, and vast soft tissue injuries resulting in treatment challenges and complications. AO/OTA classification is used frequently in classification of tibial shaft fractures. On the other hand, Gustilo-Anderson classification is preferred in open fractures [1–9]. We included both in this study.

There is a consensus in literature that, after patients' vital functions are restored and irrigation, debridement, and infection prophylaxis are achieved, primary stabilization of fracture and closing the wound as soon as possible had to be carried out. Sufficient and effective irrigation and debridement and appropriate antibiotic treatment are first steps of infection prophylaxis. While Gustilo suggests combination of first generation cephalosporins and aminoglycosides for type 3 open fractures, Zalavras et al. [20] suggest this regimen for all compound fracture types. There is not a consensus in debridement method; however, first debridement is the best chance to protect from infection and should be applied in the first six hours to keep infection rate minimum [21]. Removal of all lifeless tissues beginning from superficial layers to deeper layers is accepted as basis in open tibial fracture debridement, and Gustillo added that there should be second and even third debridement one to three days after the first. In our series, we gave combination of cephalosporin and aminoglycoside to all patients. We applied first debridement in emergency room and second debridement and soft tissue closure in operating room. Zalavras et al. [9] pointed out that wound could be closed in a way that allows drainage in type 1 and type 2 open fractures but late closure after second and even a third debridement was appropriate in type 3 fractures. In late primary closure, there are vast contamination and anaerobic infection risks in wound site. If the wound could not be closed primarily, skin graft or flap could be needed; in fact, closure has been provided better in muscle flaps and better outcomes have been achieved [22]. Also in our study, we applied soft tissue closure with loose flap in one case and with C flap in another case, and both cases were type 3B open tibial fractures. In three cases with type 3C open tibial fracture, popliteal artery was repaired in one case, posterior tibial artery was repaired in one case, and saphenous vein was repaired in another case. In definitive surgery stage radius and calcaneus fracture treatments accompanied two different type 3A open fracture surgeries.

Minimal osteosynthesis, biological fixation, and internal fixation with intramedullary nailing or external fixation with different types of fixators are used in surgical treatment of tibial compound fractures. Regardless of which treatment method is used, objective should be to obtain maximum functionality to the fractured extremity and to maintain patients' life quality with minimum damage or complication [2–4].

Use of external fixators in multiparted, defective, and contaminated open fractures, especially Gustilo-Anderson types 3B and 3C open fractures, is routinely accepted in these days [12, 23]. External fixators are frequently preferred because they are feasible, allow soft tissue treatment, provide rigid fixation, allow active axial dynamization above fracture line in early periods, and their removal is convenient. However, pin loosening rates are high and complications like malunion or nonunion are frequently seen in external fixators [23]. Gustilo-Anderson type 3B fractures may be treated with engraving locked nails too. Because these nails harm endosteal circulation least while not affecting periosteal circulation. That is why infection rates in this treatment option are found to be relatively low, but studies indicate high circulatory deficiency rates [1]. Another alternative surgical management is internal fixation following the removal of external fixator. In their meta-analysis study Bhandari et al. [10] indicated that nailing was found to have lower reoperation numbers and less superficial infection and malunion rates than external fixators and engraving nails in open tibial fractures. Gopal et al. [24] reported satisfying results when they applied minimal invasive internal fixation with biological plating in compound tibial fractures. In their review, including 11 studies, Giannoudis et al. [1] indicated that union rates were between 62 and 95%, duration of unions was between 13 and 42 weeks, reoperation rates were between 8 and 69%, and progression rate of deep infection was 11% in 492 patients who undergone plating operation for open tibial fractures.

In current studies, staged treatment options which we emphasized in our study have begun to be applied to reduce developing complications. Ma et al. [13] followed up 16 patients with compound tibial fracture (12 of them with type 3 and four of them with type 2) at least one year after two-staged protocol which comprised first stage of debridement, plating with low profile lock, and temporary external fixator and second stage of plating with definitive lock accompanied by minimal invasive percutaneus osteosynthesis and reported good results in 15 patients and bad result in one patient. In their staged treatment protocol for 10 patients with open distal tibial fractures (three with type 2, one with type 3A, and six with type 3B according to Gustilo-Anderson classification), Sohn and Kang [14] applied lateral minimally invasive plate osteosynthesis (MIPO) in second stage. Beginning treatments were debridement in first 24 hours and external fixation for six to fifty-two days. Three patients undergone bone graft after eight-week followup and no nonunion was seen after one-year followup. Superficial infections were seen in two patients and restrictions in ankle movements were seen in two patients. As a result, they indicated that their staged protocol with second stage comprising lateral MIPO was an alternative surgical option in open distal tibial fractures with its high functional recovery and low complication rate. Tong et al.'s [25] retrospective study, in which they applied two-staged treatment protocol including MIPO to 29 patients with complex pylon fractures, comprised second stage of MIPO treatment following first stage with open reduction, internal fixation, fibular fixation, and an external fixation to ankle in the first eight to twenty-four hours and soft tissue recovery for 24 to 38 days. As a result of this two-staged treatment protocol, no superficial or profound infection or problem in wound healing was seen and normal ankle function was achieved in all patients suggesting that two-staged protocol with MIPO played a key role in reduction of infection rates. As a different approach, Kim et al. [15] treated 30 patients with open proximal tibial fracture (18 patients with primary MIPO and 12 patients with staged treatment protocol including MIPO) and assessed clinical status of patients after one year of followup. Eleven patients had type 1, six patient had type 2, and 13 patients were type 3 (six were type 3A, six were type 3B, and one was type 3C). Primary union was achieved in 24 patients, and six patients undergone early bone graft. Perfect outcome was achieved in 23, and good outcome was achieved in seven patients. Three patients were superficially infected and five patients were profoundly infected but none required implant removal. As a result, infection rates were significantly lower in primary MIPO group than in staged treatment protocol group, but similar results were obtained in both groups in terms of bone union rate and functionality. Differing from others by using three-staged treatment protocol which had a first stage including debridement, antibiotherapy, and bone stabilization with external fixator, second stage of wound closure with local flap surgery and last stage of surgical bone reconstruction including bone grafting, plating, locked nailing, hybrid, monolateral external fixator, and bone dislocation with Ilizarov; Yusof and Halim [5] treated 11 patients with infected type 3B open tibial fracture. After three years of followup period, infection signs were regressed in all patients, union was achieved in nine patients, and two other patients with nonunion rejected application of other surgical treatment options. Three-staged treatment protocol was apllied in our study too. First stage was debridement, second stage included second debridement, soft tissue closure, and temporary monolateral external fixation, and third stage included circular external fixation, IMN, or definitive treatment with plating. In terms of functionality and clinical outcome, six cases were assessed as perfect, eight cases as good, two cases as medium, and three cases were assessed as bad. Results of circulary external fixation were worse than results plating or internal fixation with IMN. In our study, nonunion developed in one case, delayed union developed in another case, and osteomyelitis developed in two cases and revision surgeries were applied to these cases.

## 5. Limitations

Retrospective nature of study, small case number, and nonhomogenity of last stage of staged treatment protocol can be assessed as restrictions on the study.

## 6. Conclusion

Staged treatment option in type 3 open tibial fractures seems to be a good method in reducing complication and achieving the best result. In this protocol, temporarily monolateral external fixation and definitively plating, IMN, or Ilizarov application is usually used. Our staged treatment results were satisfactory in last stage including plating and IMN, but results of external fixation were poor. These results may be based on accompanying morbidities or properties of fractures. However, we think that definitive staged treatment protocol including internal fixation with plating or IMN of bone as soon as possible is a reliable method, especially to avoid complications as a result of external fixator and to provide patient rapport.

## Figures and Tables

**Table 1:** Patients demographics and staged treatment outcome.

Number	Age	Gender	Side	Injury	AO/OTA classification	Gustilo-Anderson grade	Comorbidity or other injuries	1st stage of treatment	2nd stage of treatment	3rd stage of treatment	Complication on followup	Outcome
1	21	F	R	FA	41A2	3B	—	DI + S	DI + EF + FCF	Ilizarov	—	Good
2	33	M	R	TA	41A2	3A	Radius fracture	DI + S	DI + EF + CRPF (for radius fracture)	Plate-screw	—	Excellent
3	15	M	R	TA	41A3	3C	Vessel injury	DI + S	DI + EF + VR	Ilizarov	Delayed union	Fair
4	38	F	L	Fall	42A1	3A	Calcaneus fracture	DI + S	DI + EF + ORIF (for calcaneus fracture)	IMN	—	Excellent
5	27	M	L	TA	42C2	3A	—	DI + S	DI + EF	IMN	—	Good
6	50	M	R	TA	42A3	3A	Hypertension	DI + S	DI + EF	IMN	—	Excellent
7	48	M	R	TA	42B2	3B	Smoke	DI + S	DİEF + FCF	Ilizarov	Nonunion	Poor
8	44	F	R	Fall	42B1	3B	Farm injury	DI + S	DI + EF	Ilizarov	Osteomyelitis	Poor
9	34	M	L	TA	42B2	3A	—	DI + S	DI + EF	IMN	—	Excellent
10	52	M	R	FA	42B3	3B	—	DI + S	DİEF + FF	IMN	—	Good
11	43	F	R	TA	42B2	3C	Vessel injury	DI + S	DİEF + VR	IMN	—	Good
12	25	M	L	TA	42C1	3B	—	DI + S	DİEF + FCF	IMN	—	Good
13	28	M	R	TA	42B2	3A	—	DI + S	DI + EF	Plate-screw	—	Excellent
14	37	M	R	Fall	42C2	3C	Vessel injury	DI + S	DI + EF + VR	Plate-screw	—	Fair
15	19	F	R	Fall	43A1	3A	—	DI + S	DI + EF	IMN	—	Good
16	66	M	R	Fall	43A2	3B	Diabetes M.	DI + S	DI + EF + FCF	Ilizarov	Osteomyelitis	Poor
17	34	M	R	TA	43A3	3B	—	DI + S	DI + EF + FF	Ilizarov	—	Good
18	32	M	L	TA	43A1	3A	—	DI + S	DI + EF	Plate-screw	—	Excellent
19	41	F	R	TA	43B1	3A	Lateral malleol Fr.	DI + S	DI + EF + ORIF (for lateral malleol fr)	Plate-screw	—	Good

DI + S: Debridement and irrigation + Splints, EF: External fixation, FCF: Fasio cutan flep, VR: Vessel repair, FF: Free flep, TA: Traffic accident, FA: Fire arms, CRPF: Closed reduction percutan fixation, ORIF: Open reduction internal fixation, IMN: Intrameduller nailing.

**Table 2:** The outcome: according to Gustilo-Anderson type or definitive surgery treatment option.

	Excellent	Good	Fair	Poor
Gustilo-Anderson Type				
Type 3A	6	3	—	—
Type 3B	—	4	—	3
Type 3C	—	1	2	—
Definitive surgery treatment				
IMN	3	5	—	—
Plate-screw	3	1	1	—
Ilizarov circular fixation	—	2	1	3
